# Supplementary material for: Electrokinetic convection-enhanced delivery for infusion into the brain from a hydrogel reservoir
Source: Commun Biol. 2024 Jul 17;7:869. doi: 10.1038/s42003-024-06404-1 (PMC11255224; doi:10.1038/s42003-024-06404-1)
Supplement: Supplementary file 2 — Supplementary Information [file 42003_2024_6404_MOESM2_ESM.pdf]

Supplementary Table 1

Experiment	Exposure time (ms)	Data_metric	Units	C Mean	C SEM	C 95%CI low	C 95%CI hi
Ex vivo	200	Distance 10%	mm	0.55	0.09	0.34	0.75
Ex vivo	200	Distance 30%	mm	0.39	0.06	0.26	0.53
Ex vivo	200	Distance 50%	mm	0.27	0.08	0.10	0.45
Ex vivo	200	Avg. Img. Intensity	Grey value (AU [0-255]) / px	0.35	0.12	0.08	0.62
Ex vivo	200	Peak height	Grey value (AU [0-255])	119.84	18.95	76.15	163.54
Ex vivo	200	Peak displacement	mm	0.17	0.03	0.10	0.23
Ex vivo	200	Area 10%	mm^2	1.29	0.42	0.33	2.26
Ex vivo	200	Area 30%	mm^2	0.11	0.07	-0.06	0.28
Ex vivo	400	Distance 10%	mm	0.73	0.11	0.48	0.99
Ex vivo	400	Distance 30%	mm	0.39	0.06	0.26	0.53
Ex vivo	400	Distance 50%	mm	0.12	0.07	-0.04	0.28
Ex vivo	400	Avg. Img. Intensity	Grey value (AU [0-255]) / px	0.85	0.25	0.28	1.42
Ex vivo	400	Peak height	Grey value (AU [0-255])	119.84	18.95	76.15	163.54
Ex vivo	400	Peak displacement	mm	0.17	0.03	0.10	0.23
Ex vivo	400	Area 10%	mm^2				
Ex vivo	400	Area 30%	mm^2				
In vivo	200	Distance 10%	mm	0.10	0.05	-0.04	0.24
In vivo	200	Distance 30%	mm	0.06	0.04	-0.06	0.17
In vivo	200	Distance 50%	mm	0.06	0.04	-0.06	0.17
In vivo	200	Avg. Img. Intensity	Grey value (AU [0-255]) / px	0.04	0.03	-0.03	0.11
In vivo	200	Peak height	Grey value (AU [0-255])	58.11	21.90	1.82	114.39
In vivo	200	Peak displacement	mm	0.05	0.02	0.00	0.10
In vivo	200	Area 10%	mm^2				
In vivo	200	Area 30%	mm^2				
In vivo	400	Distance 10%	mm	0.39	0.11	0.10	0.67
In vivo	400	Distance 30%	mm	0.10	0.06	-0.04	0.25
In vivo	400	Distance 50%	mm	0.06	0.04	-0.06	0.17
In vivo	400	Avg. Img. Intensity	Grey value (AU [0-255]) / px	0.10	0.07	-0.07	0.26
In vivo	400	Peak height	Grey value (AU [0-255])	89.57	35.57	-1.86	181.00
In vivo	400	Peak displacement	mm	0.08	0.02	0.03	0.13
In vivo	400	Area 10%	mm^2	0.35	0.21	-0.18	0.89
In vivo	400	Area 30%	mm^2	0.07	0.06	-0.08	0.21

Key

Abbreviation	Definition
C	Control
E	ECED
SEM	Standard Error of the Mean
CI	Confidence Intervals
diff	Difference: ECED - Control
stdev	Standard Deviation
df	Degrees of Freedom
tstat	T-statistic
pval	P-value, unequal variances
AU	Arbitrary Units of intensity from 0 to 255.
px	Pixel

E Mean	E SEM	E 95%CI lo	E 95%CI hi	diff Mean	diff 95%CI lo	diff 95%CI hi	tstat	df	C stdev	E stdev	pval
0.71	0.09	0.50	0.92	0.16	0.16	0.17	-1.31	16.00	0.27	0.27	0.21
0.60	0.08	0.40	0.79	0.20	0.15	0.26	-1.96	14.60	0.18	0.25	0.07
0.52	0.08	0.34	0.70	0.25	0.24	0.25	-2.27	15.99	0.23	0.24	0.04
0.75	0.14	0.42	1.08	0.40	0.34	0.46	-2.16	15.38	0.35	0.43	0.05
188.00	17.95	146.60	229.39	68.15	70.45	65.85	-2.61	15.95	56.84	53.85	0.02
0.19	0.03	0.12	0.26	0.03	0.02	0.04	-0.63	15.59	0.08	0.09	0.54
1.87	0.38	1.00	2.73	0.57	0.67	0.48	-1.02	15.82	1.25	1.13	0.32
0.44	0.18	0.03	0.84	0.33	0.10	0.56	-1.71	10.83	0.22	0.53	0.12
0.96	0.11	0.71	1.21	0.22	0.23	0.22	-1.45	16.00	0.33	0.33	0.17
0.60	0.08	0.40	0.79	0.20	0.15	0.26	-1.96	14.60	0.18	0.25	0.07
0.40	0.10	0.17	0.63	0.28	0.21	0.34	-2.28	14.47	0.21	0.30	0.04
1.58	0.27	0.95	2.22	0.73	0.67	0.80	-1.99	15.81	0.74	0.82	0.06
188.00	17.95	146.60	229.39	68.15	70.45	65.85	-2.61	15.95	56.84	53.85	0.02
0.19	0.03	0.12	0.26	0.03	0.02	0.04	-0.63	15.59	0.08	0.09	0.54
0.27	0.08	0.05	0.48	0.17	0.09	0.24	-1.66	8.58	0.13	0.21	0.13
0.16	0.07	-0.01	0.34	0.11	0.04	0.17	-1.34	8.44	0.11	0.17	0.22
0.10	0.06	-0.04	0.25	0.05	0.01	0.08	-0.66	9.28	0.11	0.14	0.52
0.12	0.03	0.03	0.21	0.08	0.06	0.11	-1.92	9.41	0.07	0.09	0.09
76.12	16.00	35.01	117.24	18.02	33.19	2.85	-0.66	9.15	53.63	39.18	0.52
0.17	0.03	0.08	0.25	0.11	0.08	0.15	-2.92	8.14	0.05	0.08	0.02
0.88	0.11	0.60	1.17	0.49	0.49	0.50	-3.14	10.00	0.27	0.27	0.01
0.33	0.07	0.14	0.52	0.23	0.18	0.27	-2.41	9.45	0.14	0.18	0.04
0.23	0.07	0.05	0.42	0.18	0.11	0.25	-2.12	8.23	0.11	0.18	0.07
0.33	0.08	0.12	0.54	0.23	0.20	0.27	-2.25	9.60	0.16	0.20	0.05
131.19	31.59	49.99	212.39	41.62	51.85	31.39	-0.87	9.86	87.12	77.37	0.40
0.21	0.03	0.12	0.29	0.13	0.09	0.17	-3.32	7.86	0.05	0.08	0.01
1.30	0.26	0.64	1.97	0.95	0.82	1.07	-2.84	9.58	0.51	0.63	0.02
0.22	0.09	0.00	0.44	0.16	0.08	0.23	-1.52	8.56	0.14	0.21	0.16
