# Supplementary material for: Electrokinetic convection-enhanced delivery for infusion into the brain from a hydrogel reservoir
Source: Commun Biol. 2024 Jul 17;7:869. doi: 10.1038/s42003-024-06404-1 (PMC11255224; doi:10.1038/s42003-024-06404-1)
Supplement: Supplementary file 3 — Description of Additional Supplementary Materials [file 42003_2024_6404_MOESM3_ESM.docx]

**Description of Additional Supplementary Files**

**File name:** Supplementary Data 1

**Description:** The numerical data for the plots in this paper.

**File name:** Supplementary Code 1

**Description:** The code for the data analysis.
