# Supplementary material for: Electrokinetic convection-enhanced delivery for infusion into the brain from a hydrogel reservoir
Source: Commun Biol. 2024 Jul 17;7:869. doi: 10.1038/s42003-024-06404-1 (PMC11255224; doi:10.1038/s42003-024-06404-1)
Supplement: Supplementary file 6 — Reporting summary [file 42003_2024_6404_MOESM6_ESM.pdf]

nature portfolio

Corresponding author(s): Jesus G. Cruz-Garza, Lokeshwar S. Bhenderu, Amir H. Faraji. DAPR. COMMSBIO-23-2176A

Last updated by author(s): Apr 22, 2024

Reporting Summary

Nature Portfolio wishes to improve the reproducibility of the work that we publish. This form provides structure for consistency and transparency in reporting. For further information on Nature Portfolio policies, see our Editorial Policies and the Editorial Policy Checklist.

Statistics

For all statistical analyses, confirm that the following items are present in the figure legend, table legend, main text, or Methods section.

n/a	Confirmed	
☐	☒	The exact sample size (n) for each experimental group/condition, given as a discrete number and unit of measurement
☐	☒	A statement on whether measurements were taken from distinct samples or whether the same sample was measured repeatedly
☐	☒	The statistical test(s) used AND whether they are one- or two-sided<br>*Only common tests should be described solely by name; describe more complex techniques in the Methods section.*
☐	☒	A description of all covariates tested
☐	☒	A description of any assumptions or corrections, such as tests of normality and adjustment for multiple comparisons
☐	☒	A full description of the statistical parameters including central tendency (e.g. means) or other basic estimates (e.g. regression coefficient) AND variation (e.g. standard deviation) or associated estimates of uncertainty (e.g. confidence intervals)
☐	☒	For null hypothesis testing, the test statistic (e.g. F, t, r) with confidence intervals, effect sizes, degrees of freedom and P value noted<br>*Give P values as exact values whenever suitable.*
☒	☐	For Bayesian analysis, information on the choice of priors and Markov chain Monte Carlo settings
☐	☒	For hierarchical and complex designs, identification of the appropriate level for tests and full reporting of outcomes
☐	☒	Estimates of effect sizes (e.g. Cohen's d, Pearson's r), indicating how they were calculated

Our web collection on statistics for biologists contains articles on many of the points above.

Software and code

Policy information about availability of computer code

Data collection	Brain Slicing in Vibratome: An NVSLM1 vibroslice was used to slice the freshly harvested brain into 500 μm coronal cross-sections for imaging.<br>Brain Imaging: A LEICA M165 FC stereoscope was used to take images of the coronal brain slices, Camera: LEICA DFC3000 G, Software: Leica Application Suite X, Leica Microsystems CMS GmbH. Bright field (BF) and DSRed (excitation band: [525-560] nm, emission band: [590-650] nm) filter were utilized to visualize the brain.
Data analysis	Image data extraction, and inter-rater reliability: [The plot profile] lines were created manually in ImageJ by heuristically starting at the surface of the brain, directing them perpendicular to the surface, along an axis where there is maximum fluorescence.<br>Statistical analyses: All statistical analyses were performed using Matlab 2022a.<br>Data and materials availability: All data associated with this study are present in the paper or the Supplementary Materials S1-3. All raw data is present Dryad, doi:10.5061/dryad.m37pvmd78.<br>Code availability: The open-source packages used in our analysis are stated and cited in Materials and Methods. Custom MATLAB scripts for analyzing the data are publicly available with the Dryad dataset at: , doi:10.5061/dryad.m37pvmd78.

For manuscripts utilizing custom algorithms or software that are central to the research but not yet described in published literature, software must be made available to editors and reviewers. We strongly encourage code deposition in a community repository (e.g. GitHub). See the Nature Portfolio guidelines for submitting code & software for further information.

Data

Policy information about availability of data

All manuscripts must include a data availability statement. This statement should provide the following information, where applicable:

- Accession codes, unique identifiers, or web links for publicly available datasets
- A description of any restrictions on data availability
- For clinical datasets or third party data, please ensure that the statement adheres to our policy

Data and materials availability: All data and code associated with this study are present in the paper or the Supplementary Materials. All raw data is present Dryad, doi:10.5061/dryad.m37pvmd78.

Research involving human participants, their data, or biological material

Policy information about studies with human participants or human data. See also policy information about sex, gender (identity/presentation), and sexual orientation and race, ethnicity and racism.

Reporting on sex and gender	N/A
Reporting on race, ethnicity, or other socially relevant groupings	N/A
Population characteristics	N/A
Recruitment	N/A
Ethics oversight	N/A

Note that full information on the approval of the study protocol must also be provided in the manuscript.

Field-specific reporting

Please select the one below that is the best fit for your research. If you are not sure, read the appropriate sections before making your selection.

☒ Life sciences ☐ Behavioural & social sciences ☐ Ecological, evolutionary & environmental sciences

For a reference copy of the document with all sections, see nature.com/documents/nr-reporting-summary-flat.pdf

Life sciences study design

All studies must disclose on these points even when the disclosure is negative.

Sample size	Sample size. The sample sizes for this study were estimated from preliminary data of ECED in hydrogel and adjusted after the initial set of experiments for ECED in-brain observations. The expected effect size was estimated as d = 2.04 based on previous experiments28 demonstrating ECED in hydrogel with similar electro-osmotic properties as the brain.. This effect size is estimated for the comparison of ECED at 25 µA and the diffusion-only control condition, 0 µA. For error probability alpha = 0.05, and Power (1 - β) = 0.80, the recommended sample size is at least N = 5 per group. Given that this preliminary study of effect size was done in hydrogel, we re-assessed the number of subjects per group with intermediate data of three subjects per group for ex vivo and in vivo experiments. The estimated effect size for ex vivo trials was d = 1.6, yielding an updated recommended sample size of N = 8 per group. The estimated effect size for in vivo trials was d = 1.8, resulting in a recommended sample size of N = 6 per group. Data collection was stopped when the specified number of subjects were obtained.
Data exclusions	Data inclusion / exclusion criteria. Experiments that were carried out to completion with no significant damage to the brain from the craniotomy procedure were included in the analysis: i.e. 30 min of the intervention with ECED or diffusion-only control at the specified experimental electrical current conditions. Experiments where the current fluctuated, due to the presence of air bubbles or other anomalies in the capillaries, were included in the analyzed results if these fluctuations were lower than the desired current (50 µA for ECED) and less than three minutes of the total intervention time. The three-minute cutoff was determined post-hoc. Thirteen out of the fourteen trials of the in vivo trials met the inclusion criteria, with one included trial presenting current fluctuation under 1 min in duration. For ex vivo trials, all eighteen samples met this inclusion criteria, with one included ECED trial presenting fluctuating current for 3 min of the total intervention duration.
Replication	All attempts for replication were successful, by following the predefined experimental design and keeping all measurement devices (e.g. imaging, annotation, etc) consistent. One section of the analysis included the measurement of fluorescence intensity along a line in the brain. This line was made by two human annotators, separately. As explained in the text: The plot profiles from both annotators were analyzed for inter-rater reliability. The mean correlation between the plot profiles was 0.98 ± 0.02 between both raters, with a slope of 1.05 ± 0.20. Discrepancies in slope were only observed in control trials, where diffusion of the fluorophore was minimal.
Randomization	The trial order of experimental conditions (ECED or control) was randomized and defined before the start of the experiment. Experiments were conducted following this pre-determined randomization.

Blinding	The animal caretakers were blinded to the allocation sequence, before and after the intervention. The experimenters were not blinded during the experiment interventions, as they had to set the current at the corresponding level for ECED or control conditions. The subject IDs after the completion of experiments were blinded to the experimental condition they were assigned to. The order of experimental conditions and subject IDs were kept in a separate data file for matching purposes at the end of data extraction. The experimenters that assessed, measured, and quantified the results were blinded to the experimental condition of the data samples. The two annotators that selected the images for analysis and created the linear intensity profiles were blinded to the experimental conditions each trial belonged to. The annotators were blinded from each other.

Reporting for specific materials, systems and methods

We require information from authors about some types of materials, experimental systems and methods used in many studies. Here, indicate whether each material, system or method listed is relevant to your study. If you are not sure if a list item applies to your research, read the appropriate section before selecting a response.

Materials & experimental systems

n/a	Involved in the study
☒	☐ Antibodies
☒	☐ Eukaryotic cell lines
☒	☐ Palaeontology and archaeology
☐	☒ Animals and other organisms
☒	☐ Clinical data
☒	☐ Dual use research of concern
☒	☐ Plants

Methods

n/a	Involved in the study
☒	☐ ChIP-seq
☒	☐ Flow cytometry
☒	☐ MRI-based neuroimaging

Animals and other research organisms

Policy information about studies involving animals; ARRIVE guidelines recommended for reporting animal research, and Sex and Gender in Research

Laboratory animals	Sprague–Dawley rat cadavers, 7-9 weeks of age were used in the ex vivo trials. These subjects were donated from a different experiment, 15-20 minutes after they were sacrificed. Exact age, weight, and sex were not available for these rat cadavers. All rats were euthanized via isoflurane and subsequently underwent a pancreatectomy. Carcasses were then placed into a plastic bag and placed at 4 ºC.<br>Sprague–Dawley rats, 8-10 weeks of age, 6 male and 6 females, were used for the in vivo trials.
Wild animals	N/A
Reporting on sex	Sex was not considered for analysis. Sex was considered for experimental design: each experimental group contained the same number of males and females.
Field-collected samples	N/A
Ethics oversight	The Institutional Animal Care and Use Committee (IACUC) at Houston Methodist Research Institute Institutional Animal Care and Use Committee approved the following procedures on protocol IS00006413.

Note that full information on the approval of the study protocol must also be provided in the manuscript.

Plants

Seed stocks	N/A
Novel plant genotypes	N/A
Authentication	N/A
